# NMDAR subunit GluN1 dynamics in urinary astrocyte-derived extracellular vesicles in anti-NMDAR encephalitis

**DOI:** 10.1016/j.bbih.2026.101381

**Published:** 2026-09-30

**Authors:** Junhua Mei, Mian-Mian Chen, Qing Yang, Shu-Xian Xu, Chao Wang, Honggang Lyu, Qian Gong, Zhongchun Liu, Edward Bullmore, Mary-Ellen Lynall, Xin-Hui Xie

**Affiliations:** a Department of Neurology, Wuhan No.1 Hospital, Wuhan, Hubei, China; b Department of Psychiatry, Renmin Hospital of Wuhan University, Wuhan, China; c State Key Laboratory of Metabolism and Regulation in Complex Organisms, Wuhan University, Wuhan, China; d Department of Psychiatry, School of Clinical Medicine, University of Cambridge, Cambridge, UK; e School of Academic Psychiatry, Institute of Psychiatry, Psychology & Neuroscience, King's College London, London, UK; f Department of Psychiatry, University of Oxford, Oxford, UK; g Sanger Institute, Wellcome Genome Campus, Hinxton, UK

**Keywords:** Exosomes, Proteomics, Urine, Auto-antibody

## Abstract

**Background and objectives:**

Longitudinal measurement *in vivo* of molecular changes in the central nervous system (CNS) is important to investigate and guide treatment of rapidly changing neurological diseases. Here, we show how a novel protocol for urinary astrocyte-derived extracellular vesicles (uADEVs) can be used non-invasively to track the dynamic trajectory of the N-methyl-D-aspartate receptor (NMDAR) subunit GluN1 in a patient with anti-NMDAR encephalitis.

**Methods:**

We collected 18 morning urine samples from the patient over 34 days of hospitalisation, as well as 15 samples from a healthy control. We isolated uADEVs, measured GluN1 concentrations, and used a wavelet transform to analyse the time-frequency dynamics of the GluN1 signal.

**Results:**

Compared with the control, GluN1 levels in the patient's uADEVs were higher on average and more variable over time. Wavelet analysis resolved (i) a low-frequency trend that decreased over time, matching the decrease of GluN1 in cerebrospinal fluid associated with clinical recovery; and (ii) a higher frequency oscillation, with GluN1 levels peaking about 48-72 hours after each of 4 cycles of methotrexate infusion.

**Conclusion:**

This case study provides preliminary evidence that urinary ADEV sampling is feasible for measuring brain biomarker dynamics linked to the clinical course and treatment of auto-immune encephalitis.

## 1 Introduction

Anti-N-methyl-D-aspartate receptor (NMDAR) encephalitis is thought to arise from antibody-induced NMDAR internalization and consequent reductions in receptor density – a mechanistic understanding primarily based on *in vitro* and animal studies (Dalmau et al., 2019; Dalmau and Graus, 2018). Direct human evidence was limited to post-mortem tissue staining until a recent positron emission tomography (PET) study provided the first such evidence *in vivo* (Galovic et al., 2023). Compared with healthy controls, patients with serum antibody-positive anti-NMDAR encephalitis showed reduced NMDAR density, whereas serum antibody-negative patients did not. However, PET has three major limitations: scans are necessarily infrequent making rapid changes during the acute stage difficult to track; the signal cannot be assigned to a specific cell type; and brain scanning is poorly tolerated in acute encephalitis.

Assays based on brain-derived extracellular vesicles (BDEVs) offer an alternative solution. These nanosized particles encapsulate proteins, lipids, and nucleic acids from intracellular cytoplasm and can cross the blood-brain barrier bidirectionally, appearing in blood. They can therefore serve as peripherally accessible proxies for the molecular status of specific brain cells (Manolopoulos et al., 2025). For example, in Alzheimer's (Jia et al., 2019) and Parkinson's disease (Jiang et al., 2021), disease-specific biomarkers measured in plasma BDEVs strongly correlate with their measurements in central nervous system (CNS)-adjacent cerebrospinal fluid (CSF).

We have recently shown that BDEVs are also measurable in urine (Xie et al., 2025). Briefly, urinary astrocyte-derived extracellular vesicles (uADEVs) were isolated using glutamate-aspartate transporter (GLAST), an astrocytic marker with 53-fold higher expression in CNS than non-CNS tissues according to the Human Protein Atlas (Uhlén et al., 2015). Total urinary EVs were first isolated, followed by immuno-capture of GLAST-positive EVs. In subsequent validation, immunogold transmission electron microscopy (TEM) demonstrated GLAST on the surface of these vesicles, which also expressed another astrocytic marker, glial fibrillary acidic protein (GFAP). Seven neurotrophic factors were enriched 23–88-fold in uADEVs compared with total urinary EVs, whereas renal markers, like sodium-chloride co-transporter (NCC) and sodium-potassium-chloride co-transporter 2 (NKCC2), which were abundant in total urinary EVs, were not detected in the uADEVs. Recent work in mice showed that EV-associated signals from intracranial tumours can reach urine through active glomerular transcytosis, although the underlying mechanisms require further investigation (Kawaguchi et al., 2026). Collectively these results indicated that EVs of specifically astrocytic origin could be isolated from urine (Xie et al., 2025).

Here, we report for the first time how our innovative protocol for uADEV isolation can be used to measure the dynamics of brain glutamatergic receptor protein in a patient over the course of treatment for anti-NMDAR encephalitis. This single case had a clear diagnosis, a dynamic disease course, and a well-regulated treatment plan, which provided a robust basis for interpreting brain molecular signals measured in uADEVs isolated from urine sampled repeatedly over time.

## 2 Case report

A 30–35-year-old female was admitted to hospital in Wuhan, China with a 12-day history of psychiatric symptoms and a 3-day history of involuntary limb movements and altered consciousness. Her initial psychiatric symptoms included auditory hallucinations (hearing voices telling her to die), recurrent replay of television images in her mind, and insomnia. She was admitted to a psychiatric hospital, where she developed fever on the second day of hospitalisation, accompanied by tremor and involuntary shaking of all four limbs. Her temperature progressively increased to a maximum of 39.8°C. Chest and head computed tomography (CT) showed no significant abnormalities. As her level of consciousness deteriorated, she was transferred to the Department of Neurology at Wuhan No. 1 Hospital. Key changes in clinical status and the timing of major treatments are shown in Fig. 1.

**Fig. 1:**
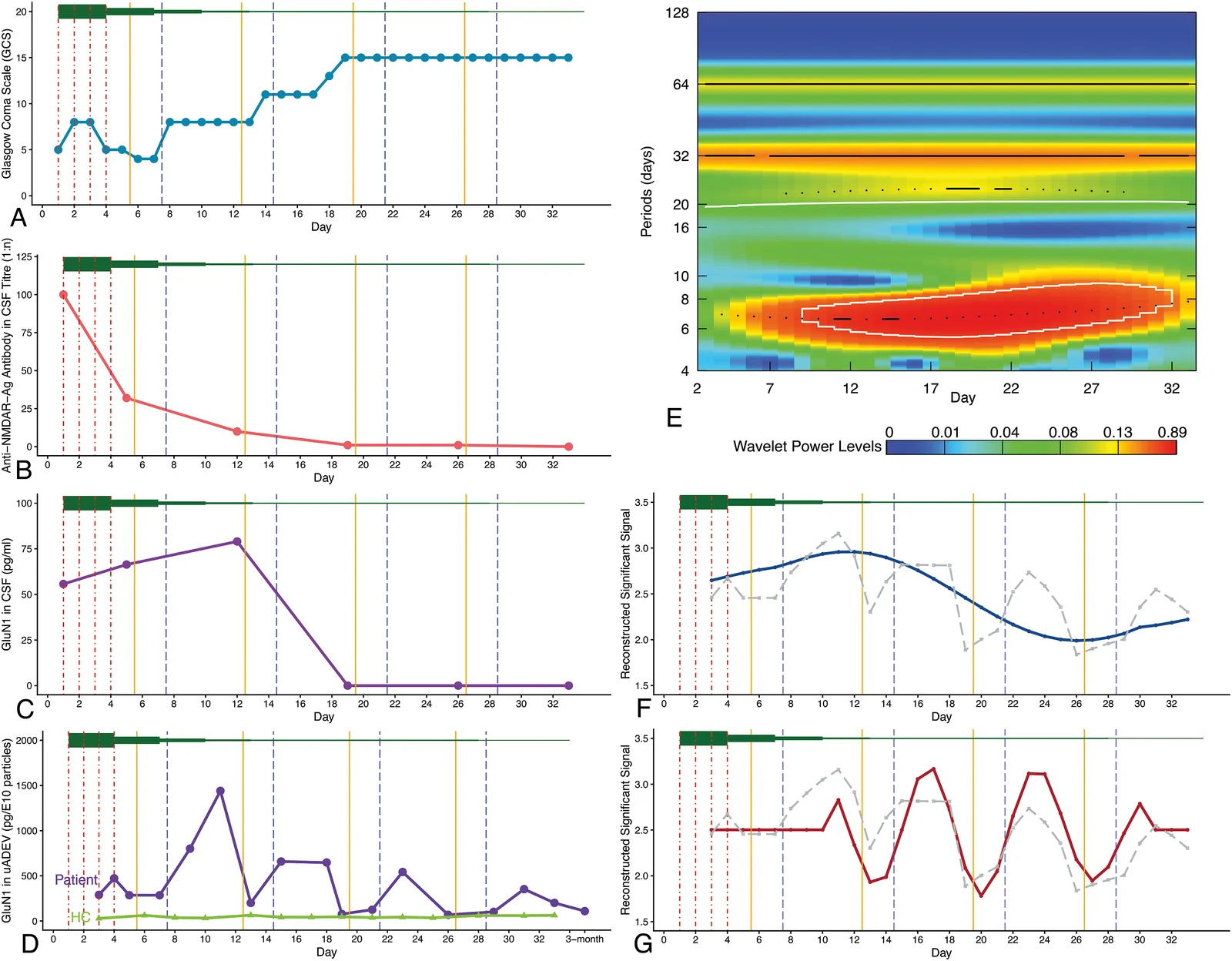
**Clinical timeline and dynamics of GluN1 measured in urinary astrocyte-derived extracellular vesicles (uADEVs) from a single case of anti-NMDAR encephalitis and a healthy control.** On admission (day 0), the patient was diagnosed with anti-NMDAR encephalitis and received four intravenous infusions of human gamma-globulin (first four red dotted vertical lines), four cycles of intrathecal methotrexate (MTX; four solid yellow vertical lines), four cycles of intravenous rituximab (four blue dashed vertical lines), and a tapering dose of methylprednisolone (green horizontal line at the top of the subfigures). These treatment indicators are the same in all subfigures. (**A**) Glasgow Coma Scale (GCS) scores (y-axis) were repeatedly measured over the course of in-patient treatment (days 0-33, x-axis), showing sustained recovery from about day 20. There was clear evidence of reduced titres of anti-NMDAR antibody (NMDAR-Ab) (**B**), and reduced concentrations of GluN1 (**C**), in six cerebrospinal fluid (CSF) samples collected over the course of treatment. We concurrently measured the concentration of GluN1 in ADEVs (pg/per 10^10^ particles by nanoparticle tracking analysis (NTA); y-axis) isolated from 18 urine samples from the encephalitis case (**D**, purple line) and compared this dynamic profile to repeated GluN1 measurements from 15 urinary ADEVs in the healthy control subject (**D**, light green line). Time-frequency decomposition by wavelet analysis of the log_10_(GluN1) time series was represented by a scalogram of variance as a function of frequency (y-axis) and time (x-axis). We interpolated values to a daily scale by averaging adjacent data points because intervals varied between 1 and 2 days (**E**). This analysis highlighted two dynamic processes: a low frequency trend to lower levels of GluN1 over the course of treatment (**F**), and a high frequency oscillation closely coupled to the timing of methotrexate infusions (**G**). The gray dashed lines in (**F**) and (**G**) represent the log_10_(ADEV GluN1) time series. (For interpretation of the references to colour in this figure legend, the reader is referred to the Web version of this article.)

On admission (Day 0), she was febrile (39.8°C) and tachycardic (115 beats/min), with a respiratory rate of 22 breaths/min and a blood pressure of 135/77 mmHg. She maintained spontaneous respiration and did not require mechanical ventilation. She was comatose, with a Glasgow Coma Scale (GCS) score of 5/15 (E1V1M3), and exhibited intermittent tremulous movements involving the perioral region and both upper limbs. Neurological examination revealed marked nuchal rigidity, a positive Kernig sign, and reduced muscle tone. Her pupils were equal in size and briskly reactive to light, and Babinski signs were absent bilaterally. Muscle strength, sensation, coordination, and extraocular movements could not be reliably assessed because of impaired consciousness. Routine CSF analysis showed a raised white cell count of 19 cells/μL (reference <5), with a lymphocytic predominance. Other routine measures were within normal limits: protein of 252 mg/L, CSF glucose was 4.0 mmol/L, with a concurrent serum glucose level of 7.8 mmol/L (CSF-to-serum glucose ratio, 0.51), an opening pressure of 145 mmH_2_O. CSF cytology showed mild lymphocytic pleocytosis with occasional activated/reactive lymphocytes. CSF anti-NMDAR antibody titre was raised at 1:100 and serum anti-NMDAR antibody was positive at a titre of 1:32, consistent with intrathecal antibody synthesis. CSF antibodies against contactin-associated protein-like 2 (CASPR2), α-amino-3-hydroxy-5-methyl-4-isoxazolepropionic acid receptor 1 (AMPA1-R), AMPA2-R, leucine-rich glioma-inactivated 1 (LGI1), γ-aminobutyric acid type B receptor (GABAB-R), and glutamic acid decarboxylase, 65-kDa isoform (GAD65) were negative. Based on symptoms and antibody titres, she was diagnosed with anti-NMDAR encephalitis (Graus et al., 2016). As treatment, she received intravenous immunoglobulin (25 g/day) on Days 1–4 of her admission (the first four red dotted vertical lines in each subfigure), intrathecal methotrexate (10 mg/day) on Days 5, 12, 19, and 26 (the four solid yellow vertical lines in each subfigure), intravenous rituximab (100 mg/day) on Days 7, 14, 21, and 28 (the four blue dashed vertical lines in each subfigure), and a tapering dose of methylprednisolone (the green horizontal line at the top of the subfigures). Methylprednisolone was started at 1000 mg/day and gradually tapered to 80 mg/day by Day 27, followed by prednisone 60 mg/day until discharge. Prednisone was subsequently tapered to 25 mg/day by the three-month follow-up. On day 22 of hospitalisation, imaging identified a 1.5 × 1.5 cm right ovarian teratoma. The teratoma was surgically removed one month after discharge. The patient's level of consciousness was assessed daily using the GCS and progressively improved from 5/15 on admission to 15/15 on Day 19, when she regained full consciousness. Her other neurological and psychiatric symptoms also improved. She was discharged on Day 34, when her CSF anti-NMDAR antibody was undetectable.

She returned to the hospital for a follow-up visit three months after discharge. At follow-up, her consciousness and daily functioning were normal, and her psychiatric symptoms and involuntary movements had resolved. CSF anti-NMDAR antibody remained negative.

The molecular target of autoantibodies in anti-NMDAR encephalitis is typically GluN1 (glutamate ionotropic receptor NMDA type subunit 1)(Dalmau et al., 2019). As methods for isolating ADEVs from urine are more mature than methods for urinary neuron-derived EVs, and because astrocytes are known to express NMDA receptors (Skowrońska et al., 2019), we predicted that urinary ADEVs could provide relevant information on the changing status of GluN1 over the course of this clinical case. During the 34 days of hospitalisation, we prospectively collected morning urine samples approximately every two days and isolated ADEVs from these 18 samples. Detailed isolation procedures are described in our previous methodological study (Xie et al., 2025). We also recruited a healthy female volunteer aged 30–35 years, collected 15 urine samples at two-day intervals, and isolated uADEVs using the same protocol. We then measured GluN1 concentrations in lysed ADEVs from the 18 patient samples, 15 control samples, and seven CSF samples from the patient, using an enzyme-linked immunosorbent assay (CUSABIO, Catalog# CSB-EL009911HU). To normalize the GluN1 concentrations, we measured the particle number of each uADEV sample using nanoparticle tracking analysis. Fig. 1 shows the complete treatment course and changes in the various biological measurements.

We hypothesized that GluN1 levels would change dynamically in relation to clinical treatment and recovery. It was evident by inspection of the GluN1 protein concentrations repeatedly measured in uADEVs (Fig. 1D) that GluN1 levels were on average higher and more variable over time in the case of encephalitis compared to the healthy control. To resolve the dynamics more precisely, GluN1 values were linearly interpolated to daily intervals, log10-transformed, and analysed using the Morlet wavelet transform for time-frequency analysis (Fig. 1E). Statistical significance was assessed using 10,000 white-noise surrogate simulations, with a significance threshold of *p* < 0.05. The wavelet transform captures how frequency content evolves over time, providing a richer picture of the signal's dynamics than time-averaged approaches like Fourier analysis. Two clear dynamics emerged: (i) a low frequency curvilinear trend from higher GluN1 concentrations in the first half of the inpatient treatment period (0-15 days) to lower levels in the second half (16-33 days) (Fig. 1F), mirroring the step-down from high CSF concentrations of GluN1 over the same period (Fig. 1C); and (ii) a high frequency oscillation which was coupled in time to the schedule of methotrexate infusions, with an upward inflection in GluN1 concentrations immediately post infusion (Fig. 1G), reaching a peak about 48h after the start of each infusion cycle. However, given the limited number of observations and the approximately two-day sampling interval, this analysis was considered exploratory and was used to describe temporal patterns rather than to provide definitive evidence of periodicity.

## 3 Discussion

It may seem counter-intuitive that increased levels of GluN1 should be associated with both increased clinical severity of this auto-immune disease and with response to its effective treatment. However, these observations can be hypothetically reconciled in terms of a simple model of endosomal, lysosomal and exosomal processing of NMDA receptors that have been compromised by anti-NMDAR auto-antibody binding or linkage.

Membrane receptors are internalised by invagination (Dalmau and Graus, 2018) of the cell surface to form membrane-bound intracellular vesicles or endosomes, which then pass through the multivesicular body (MVB) pathway, leading either to degradation by lysosomes or to extracellular release as exosomes (Scita and Di Fiore, 2010). Autoantibody binding upregulates this process nearly 8-fold (Moscato et al., 2014). Early in the treatment period, when the titre of anti-NMDA auto-antibodies was high (Fig. 1B), it is therefore predictable that there would be an increased number of endosomes being processed through the MVB pathway to be released as GluN1-enriched exosomes into the extracellular space of the brain and thence into the blood circulation and ultimately the urine.

However, the number of ADEVs measurable in the circulation does not depend solely on the rate of NMDAR internalization to form endosomes but also on the proportion of endosomes that are switched by the MVB pathway to lysosomal destruction versus exosomal release. The switch to exosomal release can be promoted by tumour protein p53 (Yu et al., 2006), a regulatory transcription factor protein that has been implicated in tumour suppression and many cell cycle processes. Methotrexate can boost p53 protein levels, peaking ∼24-48h post-dose (Huang et al., 2011), causing increased exosome production over the same period (Yu et al., 2006). Thus, the repeated cycle of increased GluN1 following methotrexate infusion could potentially be explained by the drug's known effects on p53 causing a greater proportion of GluN1-enriched endosomes to be released as exosomes, and therefore measurable in urine as ADEVs. In support of this model, it is notable that the maximum post-methotrexate level of GluN1 was typically measured in urinary ADEVs about 48-72h after dosing, i.e., it was delayed by 12-24h relative to the known methotrexate-driven peak in p53 (Huang et al., 2011; Yu et al., 2006). This time lag is approximately compatible with the time taken for brain-derived EVs to enter the bloodstream and then to be excreted in the urine.

## 4 Limitations

The limited number of measurements and approximately two-day sampling interval constrain the temporal resolution of the wavelet analysis. In addition, concurrent treatments prevent attribution of the observed GluN1 pattern specifically to methotrexate; the temporal association therefore does not establish causality. The inclusion of only one healthy control also precludes definition of a normal ADEV GluN1 reference range and the patient's deviation from this; the comparison with the control should therefore be considered descriptive. This logically coherent pattern of results from clinical investigation of a single case will clearly require replication in larger samples of patients with anti-NMDAR encephalitis, and deeper mechanistic understanding of exosome pathophysiology from future cellular and experimental medicine studies.

## 5 Conclusion

This first case report (N = 1) suggests that urinary ADEV sampling could provide a clinically acceptable and informative proxy for dynamic fluxes in astrocyte NMDAR signaling, exemplifying the diagnostic potential of this novel technique for reading “a message in a bottle” from the brain.

## CRediT authorship contribution statement

**Junhua Mei:** Writing – original draft, Supervision, Investigation, Data curation. **Mian-Mian Chen:** Investigation, Data curation. **Qing Yang:** Investigation, Data curation. **Shu-Xian Xu:** Writing – original draft. **Chao Wang:** Validation, Investigation. **Honggang Lyu:** Visualization, Validation. **Qian Gong:** Writing – original draft. **Zhongchun Liu:** Writing – review & editing, Funding acquisition. **Edward Bullmore:** Writing – review & editing, Writing – original draft. **Mary-Ellen Lynall:** Writing – review & editing, Writing – original draft. **Xin-Hui Xie:** Writing – original draft, Supervision, Formal analysis, Conceptualization.

## Ethics declaration

Written informed consent to take part in the study and to publish the article has been obtained from all participants or their legal representatives. The privacy rights of participants have been observed.

This study was performed in compliance with relevant laws, regulatory frameworks and guidelines where the research took place. This study was approved by the Ethics Committee of Wuhan No.1 Hospital. (Approval No. 2023-50)

This research follows the CARE guidelines and the CARE checklist.

## Data access statement

The data that support the findings of this study are available from the corresponding author upon reasonable request.

## Preprint availability

A preprint version of this study is available at medRxiv: https://doi.org/10.64898/2026.03.30.26349276.

## Grants

JM is supported by the Natural Science Foundation of Hubei Province (2025AFC109). ZL is funded by the National Natural Science Foundation of China (U21A20364) and the National Key R&D Program of China (2024YFC3308400). MEL and EB are supported by the ImmunoMIND hub, funded by the United Kingdom Research & Innovation (UKRI) Medical Research Council (MRC) (MR/Z50354X/1). All research at the Department of Psychiatry in the University of Cambridge is supported by the NIHR Cambridge Biomedical Research Centre (NIHR203312) and the NIHR Applied Research Collaboration East of England. MEL is part-funded by the National Institute for Health and Care Research (NIHR) Oxford Health BRC (NIHR203316).

## Declaration of competing interest

EB has received consultancy fees from Boehringer Ingelheim, Novartis, Sosei Heptares, SR One and GlaxoSmithKline; he receives book publishing royalties from Hachette and Elsevier; and he is a co-founder and stockholder of Centile Bioscience Inc. MEL receives book publishing royalties from Wiley Blackwell and Cambridge University Press. All other authors report no competing interests.

## Data Availability

The source code for this study is available at GitHub: https://github.com/xxh87/uADEV_anti_NMDAR_Encephalitis_Case.
